# Loss of chromosome Y leads to down regulation of KDM5D and KDM6C epigenetic modifiers in clear cell renal cell carcinoma

**DOI:** 10.1038/srep44876

**Published:** 2017-03-23

**Authors:** Madeleine Arseneault, Jean Monlong, Naveen S. Vasudev, Ruhina S. Laskar, Maryam Safisamghabadi, Patricia Harnden, Lars Egevad, Nazanin Nourbehesht, Pudchalaluck Panichnantakul, Ivana Holcatova, Antonin Brisuda, Vladimir Janout, Helena Kollarova, Lenka Foretova, Marie Navratilova, Dana Mates, Viorel Jinga, David Zaridze, Anush Mukeria, Pouria Jandaghi, Paul Brennan, Alvis Brazma, Jorg Tost, Ghislaine Scelo, Rosamonde E. Banks, Mark Lathrop, Guillaume Bourque, Yasser Riazalhosseini

**Affiliations:** 1 Department of Human Genetics, McGill University, 1205 Dr Penfield Avenue, Montreal, QC, H3A 1B1, Canada; 2 McGill University and Genome Quebec Innovation Centre, 740 Doctor Penfield Avenue, Montreal, QC, H3A 0G1, Canada; 3 Leeds Institute of Cancer and Pathology, University of Leeds, Cancer Research Building, St James’s University Hospital, Leeds, LS9 7TF, UK; 4 International Agency for Research on Cancer (IARC), 150 cours Albert Thomas, 69008 Lyon, France; 5 Karolinska Institutet, Department of Pathology, SE-171 77 Stockholm, Sweden; 6 First Faculty of Medicine, Institute of Hygiene and Epidemiology, Charles University in Prague, Studničkova 7, Praha 2, 128 00 Prague, Czech Republic; 7 University Hospital Motol, V Úvalu 84, 150 06 Prague, Czech Republic; 8 Department of Preventive Medicine, Faculty of Medicine, Palacky University, Hnevotinska 3, 775 15 Olomouc, Czech Republic; 9 Department of Cancer Epidemiology and Genetics, Masaryk Memorial Cancer Institute and MF MU, Zluty Kopec 7, 656 53 Brno, Czech Republic; 10 National Institute of Public Health, Dr Leonte Anastasievici 1–3, sector 5, Bucuresti 050463, Romania; 11 Carol Davila University of Medicine and Pharmacy, Th. Burghele Hospital, 20 Panduri Street, 050659 Bucharest, Romania; 12 Russian N.N. Blokhin Cancer Research Centre, Kashirskoye shosse 24, Moscow 115478, Russian Federation; 13 European Molecular Biology Laboratory, European Bioinformatics Institute, EMBL-EBI, Wellcome Trust Genome Campus, Hinxton, CB10 1SD, UK; 14 Laboratory for Epigenetics & Environment, Centre National de Génotypage, CEA-Institut de Génomique, 2 rue Gaston Crémieux, 91000 Evry, France

## Abstract

Recent genomic studies of sporadic clear cell renal cell carcinoma (ccRCC) have uncovered novel driver genes and pathways. Given the unequal incidence rates among men and women (male:female incidence ratio approaches 2:1), we compared the genome-wide distribution of the chromosomal abnormalities in both sexes. We observed a higher frequency for the somatic recurrent chromosomal copy number variations (CNVs) of autosomes in male subjects, whereas somatic loss of chromosome X was detected exclusively in female patients (17.1%). Furthermore, somatic loss of chromosome Y (LOY) was detected in about 40% of male subjects, while mosaic LOY was detected in DNA isolated from peripheral blood in 9.6% of them, and was the only recurrent CNV in constitutional DNA samples. LOY in constitutional DNA, but not in tumor DNA was associated with older age. Amongst Y-linked genes that were downregulated due to LOY, *KDM5D* and *KDM6C* epigenetic modifiers have functionally-similar X-linked homologs whose deficiency is involved in ccRCC progression. Our findings establish somatic LOY as a highly recurrent genetic defect in ccRCC that leads to downregulation of hitherto unsuspected epigenetic factors, and suggest that different mechanisms may underlie the somatic and mosaic LOY observed in tumors and peripheral blood, respectively.

Chromosomal aneuploidy is a common phenomenon in many cancers, and the analysis of copy number variations (CNVs) across multiple samples has helped identify relevant driver genes for human cancers. For example, several oncogenes including *MYC, EGFR, ERBB2* and *CCND1* are recurrently amplified through chromosomal or focal gains, while multiple tumor suppressors such as *ATM, PTEN* and *CDKN2A* are commonly deleted in different cancers^1^.

Clear cell renal cell carcinoma (ccRCC), which accounts for 75–80% of all renal cell carcinomas, is characterized by loss of chromosome 3p in about 90% of the sporadic cases^2^. Remarkably, 3p harbors the four most commonly mutated genes in ccRCC whose cancer-driving activities have been established in the disease; *VHL*^3^, *PBRM1*^4^, *SETD2*^5^, and *BAP1*^6^, which are mutated in 80%, 40%, 19% and 12% of cases, respectively^7^,^8^,^9^. Inactivation of VHL leads to constitutive stabilization of the hypoxia inducible transcription factors (HIF), and abnormal activation of their downstream genes, which contribute to cancer development^10^. The remaining three genes encode proteins involved in chromatin remodeling and histone modifications, highlighting the important role of epigenome aberration in the disease^2^. While the incidence of ccRCC is increasing worldwide, the male-to-female incidence ratios are typically within the range of 1.5–2:1.0^11^, arguing for a sex-specific analysis of the genomic abnormalities. Here, we set out to investigate the occurrence and the extent of germline and somatic CNVs in sporadic ccRCC in male and female patients separately, and to further characterize those affecting sex chromosomes.

## Results and Discussion

### Loss of chromosome Y is common in ccRCC

**Figure 1:**
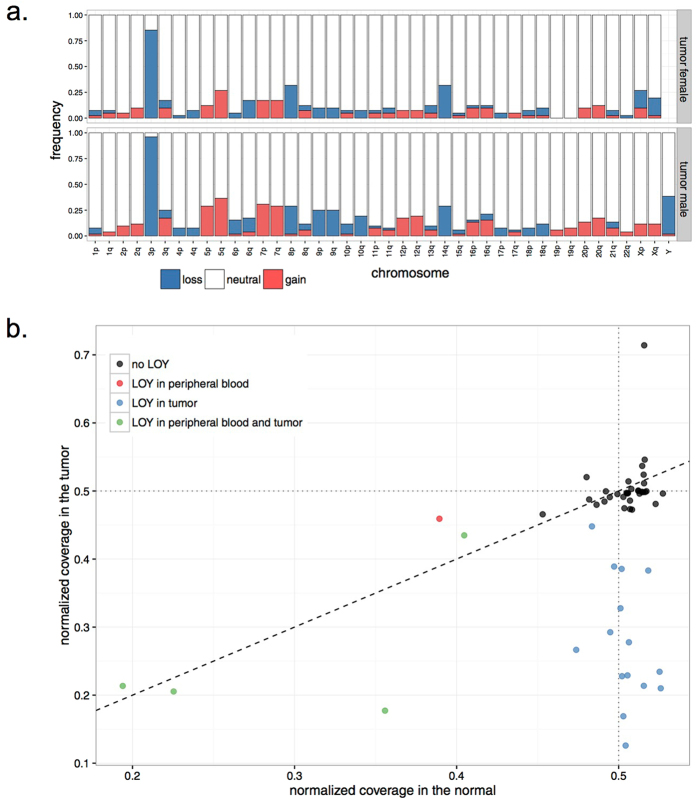
Copy number analysis in ccRCC. (**a**) Bar graphs show the frequency of copy number variations across the genome in ccRCC tumors. Frequencies are presented in samples from female and male cases separately. (**b**) Status of chromosome Y in DNA isolated from tumors (Y-axis) and patient-matched peripheral blood (X-axis) is shown for individual male subjects. In samples affected by LOY, the normalized coverage of chromosome Y, shown on Y and X axes for tumor and normal samples, respectively, is lower than the expected value of 0.5. The color codes define patient groups with different states for LOY.

Using whole-genome sequencing (WGS) data of ccRCC and matched constitutional DNA sample pairs, which we have reported recently^12^, we interrogated CNVs in DNA from 52 male and 41 female patients (discovery set; Supplementary Table 1) by analyzing coverage of sequencing reads mapped to each chromosome (see Methods). In line with previous literature, the most frequent somatic CNV was the loss of 3p detected in 91% of samples, followed by recurrent gains of chromosomes 5q (32%), 7 (23.6%), 12 (13%), and losses of chromosomes 14q (30%), 8p (29%) and 9 (16%). Overall, tumors from male patients exhibited higher prevalence for the recurrent chromosomal aberrations, in particular for gain of 7q (28% in males vs. 17% in females) and deletion of 9p (25% in males vs. 10% in females) (Fig. 1a). In contrast, we observed that loss of chromosome X (LOX) exclusively happens in female patients (17.1% of female cases). Given that several X-linked genes escape X-inactivation, and have therefore two functional copies in females but one in males, this observation suggests that presence of a copy of chromosome X may potentially be essential for the survival of cancer cells. Curiously, whereas no tumors from male patients displayed LOX, loss of chromosome Y (LOY) was the second most frequent somatic chromosome aneuploidy in these tumors (36.5% of male subjects, N = 19; Fig. 1a). The fraction of cells estimated to be affected by somatic LOY in these patients ranged from 11% to 75%, and in 14 patients somatic LOY was detected in at least 20% of the cells (Fig. 1b). Next, we examined the presence of CNVs in constitutional DNA isolated from peripheral samples collected from the same patients. Of significance, LOY was the only recurrent aneuploidy in constitutional DNA of our samples that was detected in 5 male patients (9.6%; Supplementary Figure 1), of which 4 showed the deletion in more than 20% of cells (Fig. 1b). Corroborating previous studies^13^,^14^, the observed LOY was associated with older age in patients (*P* = 0.04); the average age of patients with LOY in the peripheral blood was 68.9 year in comparison to 58.8 year in those without this abnormality. Notably, we did not observe any association between age of patients and extent of somatic LOY in tumors of the affected patients.

### LOY is a whole-chromosome event

**Figure 2:**
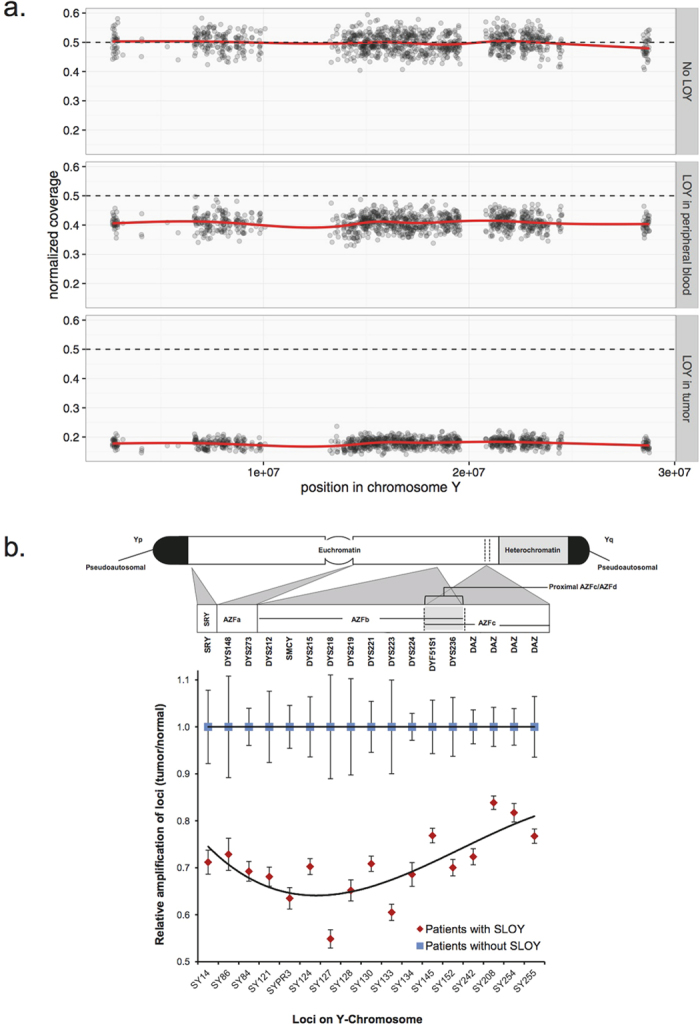
LOY affects whole chromosome. (**a**) Sequencing coverage across chromosome Y is shown in constitutional DNA samples without (top) and with LOY (middle), and in a tumor sample with LOY (bottom). (**b**) The cartoon on top depicts the location of the loci examined by PCR on Y chromosome. The dot graph on bottom shows average of relative amplification values (Tumor/normal samples of the same patient) for each locus in patients with (red) and without (blue) somatic LOY (SLOY). Error bars show the range across patients of each group.

Given the high prevalence of LOY in tumors and peripheral DNA of male patients, we further analyzed LOY in our sample series, particularly whether the observed LOY spans the whole chromosome or is focal. Analysis of sequencing read coverage along chromosome Y showed that the loss is observed throughout the chromosome in samples affected by LOY (Fig. 2a), suggesting that the deletion affects the whole chromosome. Based on availability of DNA, we subjected samples from seven of the patients affected with somatic LOY to verification by an orthogonal Y-Chromosome deletion detection assay surveying the presence of twenty specific regions of the Y chromosome by polymerase chain reaction (PCR) (see Methods). Somatic LOY at the chromosomal level was confirmed in all examined tumors, evident from an attenuated amplification of Y-chromosome-specific loci in DNA isolated from tumor samples compared to that of the matched constitutional DNA. This pattern was not observed in samples of other male patients who had not been identified as being affected by somatic LOY based on the analysis of their WGS data (Fig. 2b).

To confirm these findings, we screened tumor and matched control DNA sample pairs of an additional 48 male ccRCC patients (validation set) for LOY using the above PCR-based assay. This analysis revealed somatic LOY in 20 (42.7%) of the validation sample set, demonstrating that this is a common genomic aberration in ccRCC, detected in 39.6% overall (discovery and validation sets; n = 100) of male ccRCC patients (Supplementary Figure 2). Analysis of association between somatic LOY and clinical annotations including tumor stage or grade did not show any significant relationships.

### LOY results in downregulation of epigenetic modifier genes

**Figure 3:**
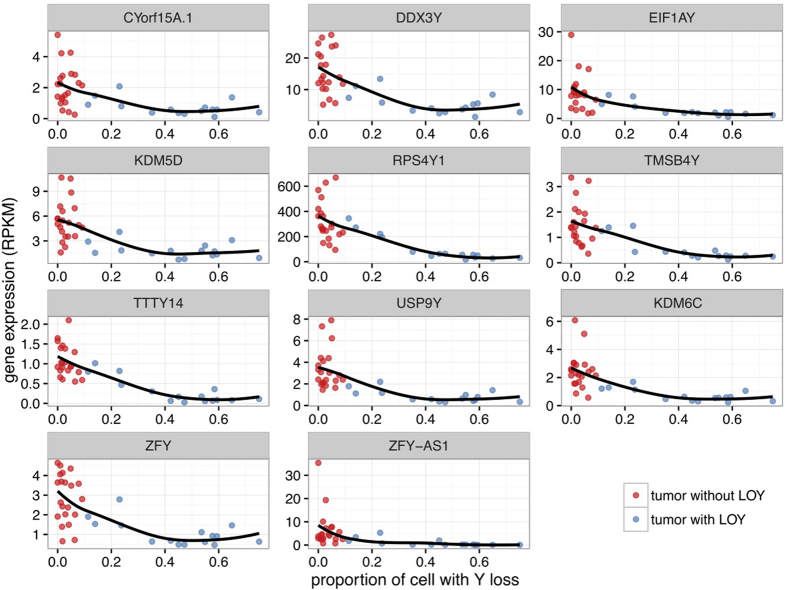
Somatic LOY leads to downregulation of Y-linked genes. Expression of Y chromosome genes downregulated in patients affected by somatic LOY is compared to the proportion of cells estimated to harbor somatic LOY in individual tumor samples.

We further examined the possible effect of somatic LOY at the RNA level by interrogating a RNA-Seq dataset on gene expression in normal and tumor samples from male patients within the discovery set^12^. We found that 11 genes had significantly different patterns of expression in tumors of the patients with and without somatic LOY (false-discovery rate (FDR) <0.01; Supplementary Table 2). These 11 genes were located on chromosome Y, and while expressed in normal kidney tissue, exhibited lower expression in tumors of patients harboring somatic LOY, indicating that this aberration may have functional consequences through deregulation of the affected genes. Moreover, the level of expression of each gene was found to be inversely correlated to the proportion of cells affected by LOY (Fig. 3). This observation was confirmed using gene expression data generated by microarrays, which was available for 29 tumors of the validation set^15^ (Supplementary Figure 3). We surveyed the list of genes affected for potential functionally-relevant candidates. Among these genes, *TMSB4Y* has recently been identified as a tumor suppressor gene downregulated in male breast cancers^16^, but not connected to ccRCC. Likewise, deletion of *KDM5D* has been detected in 52% of prostate cancers^17^. *KDM5D* encodes a lysine-specific histone H3 demethylase, which plays an important role in epigenetic regulation^18^. Furthermore, it has been shown that knockdown of KDM5D through RNA-interference (RNAi) increases cell proliferation and reduces apoptosis in prostate cancer^19^, suggesting a tumor suppressor function for this gene. Intriguingly, *KDM5C*, the X-linked homologue of *KDM5D* is recurrently mutated in ccRCC^8^,^9^,^12^, and its inactivation leads to genomic instability in ccRCC through deregulation of H3K4 methylation^20^. *KDM5D* shows 85% sequence identity to *KDM5C,* and the products of these two genes possess a similar function in demethylating tri-methyl H3K4^18^,^20^. Given this functional similarity, we surveyed the mutational status of *KDM5C* in our discovery set, and investigated possible relationships between mutational status of *KDM5C* and *KDM5D* in tumors of male patients. In female patients, *KDM5C* was deleted in tumors of 7 cases through somatic LOX, and was affected by focal somatic deletions in two additional patients. Furthermore, somatic mutations of *KDM5C* were present in tumors of 3 patients who were also affected with LOX (*P* = 0.003, Fisher’s exact test, Supplementary Figure 4). Overall, *KDM5C* was affected with somatic genomic aberrations in 9 out of 41 (22%) female cases. As *KDM5C* escapes the X-inactivation^21^, the concomitant mutations of *KDM5C* and LOX in the same tumors may suggest that this gene is a classical tumor suppressor affected with bi-allelic inactivation in ccRCC. In male cases, we identified *KDM5C* mutations in tumors of 3 patients (5.8%), of which one was also affected by somatic LOY (Supplementary Figure 4). We did not detect any mutation or a focal CNV affecting *KDM5D* in tumors of the male patients who did not exhibit LOY.

Our list of LOY-associated down-regulated genes (Supplementary Table 2) includes another epigenome modifier with an X-linked homologue that is also recurrently mutated in ccRCC; *UTY/KDM6C*. KDM6C demethylates H3K27, a function similar to that of KDM6A^22^. These genes also share over 83% in sequence similarity, resulting in highly conserved active sites in their products. Mutations of *KDM6A* leading to its inactivation have been recurrently observed in ccRCC^8^,^23^, highlighting this gene as a potential key tumor suppressor in renal cancer. In addition to being affected by somatic LOX in 7 female patients, *KDM6A* was also affected by focal deletion in a female patient in our cohort.

### KDM5D expression reduces viability of renal cancer cells

**Figure 4:**
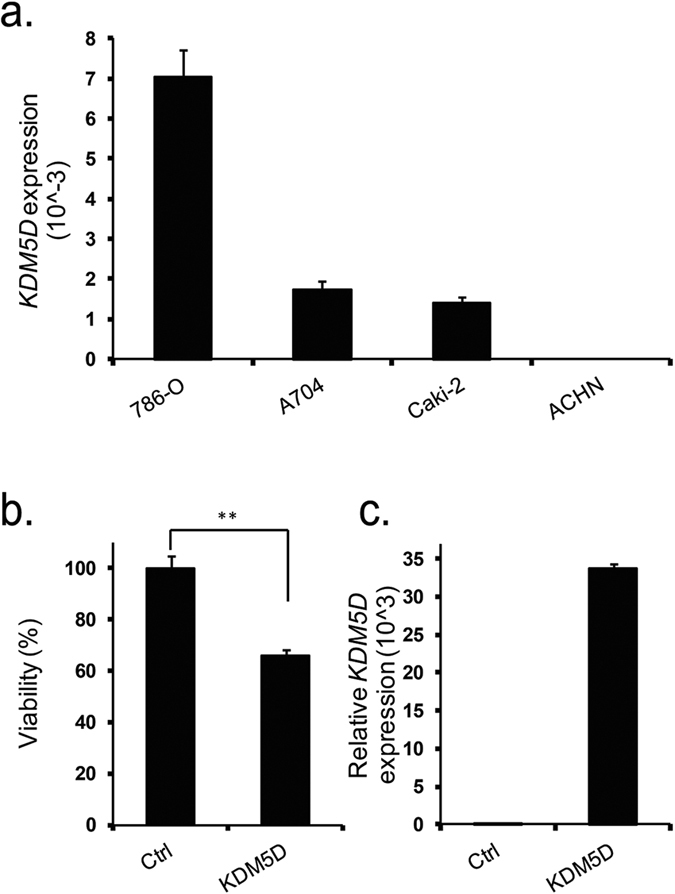
Effect of KDM5D on viability of renal cancer cells. (**a**) Expression levels of KDM5D mRNA in renal cancer cell lines derived from tumors procured from male patients, as measured by qRT-PCR. *GAPDH* served as a housekeeping gene for measurement of relative gene expression. (**b**) Over expression of KDM5D in ACHN cell line reduces cell viability. Values are the mean ± SD of six independent experiments. **P < 0.01 when compared to the corresponding results from control (ctrl) (Mann-Whitney U test). (**c**) Over expression of KDM5D following transfection was confirmed using qRT-PCR.

Given the reported tumor-suppressive function of KDM5D in prostate cancer^19^, and of its X-link homolog KDM5C in renal cancer^20^, we set out to examine whether KDM5D expression has an anti-tumor activity in renal cancer. We first evaluated KDM5D expression levels in several renal cancer cell lines, which have been derived from tumors resected from male patients. Amongst cell lines examined, ACHN cell line did not show any expression for KDM5D (Fig. 4a). This observation was in line with a previous study reporting the loss of chromosome Y in ACHN cell line^24^. We therefore selected this cell line for functional analysis of KDM5D expression. Ectopic expression of KDM5D cells reduced cell viability to 65% as compared to control transfection (Fig. 4b,c), suggesting the potential involvement of KDM5D depletion in renal cancer pathology.

## Conclusions

Emerging data emphasizes an association between LOY in peripheral blood and higher risk of cancer^25^. Likewise focal or chromosome-level somatic LOY occurs recurrently in different malignancies; however, current knowledge of mechanisms by which LOY may contribute to cancer is limited. Recent genomic studies of ccRCC have highlighted the importance of molecular aberrations that impair the function of chromatin remodeling and epigenetic modifiers in ccRCC development^5^,^20^,^26^,^27^,^28^,^29^. Our study expands these findings by highlighting the prevalence of somatic LOY among men affected by ccRCC, and suggesting a functional relevance for this aberration through down-regulation of previously unrecognized epigenetic modifiers *KDM5D* and *KDM6C*. Given the functional similarities between these genes and their X-linked homologs, it is plausible that down-regulation of *KDM5D* and *KDM6C*, through somatic LOY, may contribute to ccRCC development or progression. Our *in vitro* data shows that over expression of KDM5D in cancer cells that are affected by LOY reduces cell viability. These findings indicate that down-regulation of KDM5D through LOY may contribute to the pathogenesis of renal cancer. However, further detailed analysis through future functional studies is warranted to understand the exact function and pathway context of KDM5D in renal cancer.

## Methods

### Patient samples and DNA isolation

Clinical information for patients included in this study is presented in Supplementary Table 1. Patients undergoing nephrectomy for suspected renal cancer during the period December 2008 to March 2011 at St James’s University Hospital in Leeds, UK; University Hospital Motol, Prague, Czech Republic; Masaryk Memorial Cancer Institute, Brno, Czech Republic; Th. Burghele Hospital, Bucharest, Romania; and N. N. Blokhin Cancer Research Centre, Moscow, Russia, were recruited to the study after informed consent was obtained. Recruitment in Central and Eastern Europe was coordinated by the International Agency for Research on Cancer (IARC). All experiments and methods were performed in accordance to the ethics guidelines from the International Cancer Genome Consortium (ICGC) and to the relevant national regulations and with sampling and clinical data collection being undertaken according to predefined standard operating procedures (SOPs) based on guidelines from ICGC. Ethical approvals were obtained from the Leeds (East) Local Research Ethics Committee, the IARC Ethics Committee, as well as from local ethics committee for recruiting centers in Czech Republic, Romania, and Russia. DNA from fresh-frozen tumor tissue samples and buffy coat was isolated using Autopure (Qiagen) as described previously^12^, and were quantified by Quant-iT PicoGreen dsDNA Assay Kit (Invitrogen, ON, CAN).

### Inference of LOY from WGS data

WGS data of tumor and blood DNA samples studied here were reported previously^12^. To detect aneuploidy and LOY from WGS data, we first measured read coverage across the genome in 5 Kbp bins. In each sample, the coverage was normalized by the median coverage across the autosomes. We then estimated, for each sample, the median normalized coverage in each chromosome arm. The only exception was chromosome Y which was considered as a whole. In order to avoid noise due to mappability issues, we used only the top 1000 bins with the lowest median divergence from the expected baseline in the normal samples. We used this normalized median coverage per chromosome arm to test aneuploidy in each sample. For each chromosome arm (or chromosome Y), a mixture of two Gaussian distributions was fitted to the empirical distribution of the median normalized coverage across samples. The main Gaussian was used as the null distribution (Supplementary Figure 5) to derive P-values. A chromosome arm was flagged as aneuploid if the Bonferonni-adjusted P-value was smaller than 0.01 and at least 10% of cells were affected. The proportion of cell with aneuploidy was estimated as the proportion of missing/excess coverage. For LOY, we expect a normalized coverage of 0.5 and the proportion of cells with LOY was (0.5-coverage)/0.5.

We used a logistic regression to test the association of LOY with age. Finally, the CNVs used for *KDM5C* or *KDM6A* deletion investigation were detected by PopSV^30^ using the normal samples as reference and 5 Kbp bins.

### PCR-based detection of LOY

To examine the status of LOY in DNA of tumor and blood samples, Y Chromosome Deletion Detection System assay, Version 2 (Promega, WI, USA) was used as instructed by the manufacturer. Briefly, 20 specific regions of the Y chromosome were amplified by PCR using 5 multiplex master mixes, and PCR products were loaded on a QIAxcel instrument (Qiagen, ON, CAN). Densities of PCR products were estimated by BioCalculator software (v.3.2) and a normalization was performed by the control primer pair included in each multiplex master mix to control the amplification efficacy. We also included samples from three male subjects without LOY and one female sample to control the performance of the assay. Similar to the analysis on WGS data, the probes were first normalized by the median probe amplification value across the normal samples. Then the median of the normalized amplification was computed for each sample. It summarized the overall amplification of chromosome Y in each sample. These values were used to produce Supplementary Figure 2 and to identify LOY. Following the same analysis as for the WGS data, the mixture of Gaussian distributions was fitted on the normalized amplification of the normal samples. Samples which deviated significantly (*P* < 0.01) from the expected amplification and with an estimated proportion of affected of cells >10% were flagged as being affected by LOY.

### Gene expression analysis

Transcriptome profiles of the tumor samples included in this study (previously reported in our earlier publication^12^), were used to examine differential gene expression between male subjects affected with somatic LOY and those without this abnormality. RNA-seq data was available for tumors of 34 patients, of which 21 had RNA-seq for matched normal kidney samples. Differentially expressed genes between tumors affected with somatic LOY and those without this abnormality were identified using Student’s T-Test on log2-transformed RPKM data, and the Benjamini-Hochberg method was used to correct multiple testing. Genes with a FDR< 0.01 were considered differentially expressed. A linear regression was used to test the association between the proportion of cells with somatic LOY and gene expression (RPKM).

Gene expression microarray data for 29 tumors of validation samples had previously been reported^15^, and were used to confirm the anti-correlation between the proportion of cells with somatic LOY and gene expression levels (log2 intensity).

### Cell viability assay

Renal cancer cell lines 786-O, A704, Caki-2, ACHN were obtained from ATCC (Rockville, USA) and cultured in RPMI, EMEM and McCoy medium supplemented with 10% (v/v) fetal bovine serum (FBS), 100 U/ml penicillin and 100 lg/ml streptomycin. Cells were incubated at 37 °C and 5% (v/v) CO2. For viability assays, 5000 cells were transfected with 100 ng of either KDM5D cDNA-expressing (courtesy of Dr. Stephane Richard) or control empty vector (Sigma, Oakville, Canada) in 96-well plates using Lipofectamine 3000 (Invitrogen) according to the manufacturer’s instructions. CellTiter-Glo assay (Promega, WI, USA) was used to assess cell viability after 72 hours post-transfection.

### Quantitative real-time PCR (qRT-PCR)

Total RNA was extracted from cells using miRNeasy kit (Qiagen, Toronto, Canada) according to the supplier protocols. 1 μg RNA was reverse transcribed into complementary DNA (cDNA) using Transcriptor First Strand cDNA Synthesis Kit (Roche, Laval, Canada) following instructions provided by the manufacturer. Real-time PCR reactions were prepared using LightCycler 480 SYBR green I master kit (Roche), and were run on a LightCycler 480 instrument (Roche) according to the manufacturer’s recommendations. Triplicate PCR reactions were performed for each sample to ensure reliability. Expression of *KDM5D* mRNA was normalized to the expression of the housekeeping gene *GAPDH*, and was reported as 2^−ΔCt^. All the primers were purchased from IDT (Coralville, IA, US). The sequences of primers were CGTGGAAGGACTCATGACCA (GAPDH forward), GCCATCACGCCACAGTTTC (GAPDH reverse), CGCAGCTTTGAAGAGCTAAG (KDM5D forward) and CAGCTGTGGAGTGTCCATCC (KDM5D reverse).

## Additional Information

**How to cite this article:** Arseneault, M. *et al*. Loss of chromosome Y leads to down regulation of KDM5D and KDM6C epigenetic modifiers in clear cell renal cell carcinoma. *Sci. Rep.* **7**, 44876; doi: 10.1038/srep44876 (2017).

**Publisher's note:** Springer Nature remains neutral with regard to jurisdictional claims in published maps and institutional affiliations.

## Supplementary Material

Supplementary Information
