# Supplementary material for: Loss of chromosome Y leads to down regulation of KDM5D and KDM6C epigenetic modifiers in clear cell renal cell carcinoma
Source: Sci Rep. 2017 Mar 23;7:44876. doi: 10.1038/srep44876 (PMC5362952; doi:10.1038/srep44876)

Loss of chromosome Y leads to down regulation of KDM5D and KDM6C epigenetic modifiers in clear cell renal cell carcinoma

Madeleine Arseneault[1,2#], Jean Monlong[1,2#], Naveen S. Vasudev[3], Ruhina S. Laskar[4], Maryam Safisamghabadi[1,2], Patricia Harnden[3], Lars Egevad[5] , Nazanin Nourbehesht[1,2], Pudchalaluck Panichnantakul[1,2], Ivana Holcatova[6], Antonin Brisuda[7], Vladimir Janout[8], Helena Kollarova[8], Lenka Foretova[9], Marie Navratilova[9], Dana Mates[10], Viorel Jinga[11], David Zaridze[12], Anush Mukeria[12], Pouria Jandaghi[1,2], Paul Brennan[4], Alvis Brazma[13], Jorg Tost[14], Ghislaine Scelo[4], Rosamonde E Banks[3], Mark Lathrop[1,2], Guillaume Bourque[1,2], Yasser Riazalhosseini[1,2,*]

[1] Department of Human Genetics, McGill University, 1205 Dr Penfield Avenue, Montreal, QC, H3A 1B1, Canada
[2] McGill University and Genome Quebec Innovation Centre, 740 Doctor Penfield Avenue, Montreal, QC, H3A 0G1, Canada
[3] Leeds Institute of Cancer and Pathology, University of Leeds, Cancer Research Building, St James's University Hospital, Leeds, LS9 7TF, UK
[4] International Agency for Research on Cancer (IARC), 150 cours Albert Thomas, 69008 Lyon, France
[5] Karolinska Institutet, Department of Pathology, SE-171 77 Stockholm, Sweden
[6] First Faculty of Medicine, Institute of Hygiene and Epidemiology, Charles University in Prague, Studničkova 7, Praha 2, 128 00 Prague, Czech Republic.
[7] University Hospital Motol, V Úvalu 84, 150 06 Prague, Czech Republic.
[8] Department of Preventive Medicine, Faculty of Medicine, Palacky University, Hnevotinska 3, 775 15 Olomouc, Czech Republic
[9] Department of Cancer Epidemiology and Genetics, Masaryk Memorial Cancer Institute and MF MU, Zluty Kopec 7, 656 53 Brno, Czech Republic
[10] National Institute of Public Health, Dr Leonte Anastasievici 1–3, sector 5, Bucuresti 050463, Romania
[11] Carol Davila University of Medicine and Pharmacy, Th. Burghele Hospital, 20 Panduri Street, 050659 Bucharest, Romania
[12] Russian N.N. Blokhin Cancer Research Centre, Kashirskoye shosse 24, Moscow 115478, Russian Federation
[13] European Molecular Biology Laboratory, European Bioinformatics Institute, EMBL-EBI, Wellcome Trust Genome Campus, Hinxton, CB10 1SD, UK
[14] Laboratory for Epigenetics & Environment, Centre National de Génotypage, CEA-Institut de Génomique, 2 rue Gaston Crémieux, 91000 Evry, France

[#] These authors contributed equally to this work.

* Correspondence should be addressed to: Y. Riazalhosseini: yasser.riazalhosseini@mcgill.ca

Supplementary Tables

Supplementary Table S1. Characteristics of patients included in the study

Characteristics	Discovery set				Validation set (males only)		Total (males)	
	Females		Males					
	N	*%*	N	*%*	N	*%*	N	*%*
**Tumor grade**								
1	1	*2.4*	1	*1.9*	3	*6.3*	4	*4.0*
2	28	*68.3*	26	*50.0*	23	*47.9*	49	*49.0*
3	5	*12.2*	13	*25.0*	17	*35.4*	30	*30.0*
4	7	*17.1*	12	*23.1*	5	*10.4*	17	*17.0*
**Tumor stage**								
I	27	*65.9*	24	*46.2*	29	*60.4*	53	*53.0*
II	5	*12.2*	4	*7.7*	1	*2.1*	5	*5.0*
III	5	*12.2*	15	*28.8*	6	*12.5*	21	*21.0*
IV	4	*9.8*	9	*17.3*	10	*20.8*	19	*19.0*
NA	0	*0.0*	0	*0.0*	2	*4.2*	2	*2.0*
**Country of residence**								
Czech Republic	8	*19.5*	20	*38.5*	23	*47.9*	43	*43.0*
Romania	5	*12.2*	8	*15.4*	0	*0.0*	8	*8.0*
Russia	15	*36.6*	8	*15.4*	18	*37.5*	26	*26.0*
UK	13	*31.7*	16	*30.8*	7	*14.6*	23	*23.0*
**Age at surgery:** median (range)	62 (39 - 83)		60 (40 - 79)		56 (38 - 74)		58 (38 - 79)	
**Total**	**41**		**52**		**48**		**100**	

Supplementary Table S2. Genes differentially expressed between tumors with and without somatic LOY. Fold-change of differential expression between the two tumor sets.

Gene	chromosome	Fold-change (Differential expression)
KDM5D	Y	-1.3
USP9Y	Y	-1.4
ZFY	Y	-1.1
UTY/KDM6C	Y	-1.2
NLGN4Y	Y	-0.8
DDX3Y	Y	-1.8
EIF1AY	Y	-1.8
TMSB4Y	Y	-0.9
RPS4Y1	Y	-2.6

Supplementary Figures

Supplementary Figure S1. Copy number analysis in peripheral blood. Bar graphs show the frequency of copy number variations across the genome in peripheral blood. Frequencies are presented in samples from female and male cases separately.

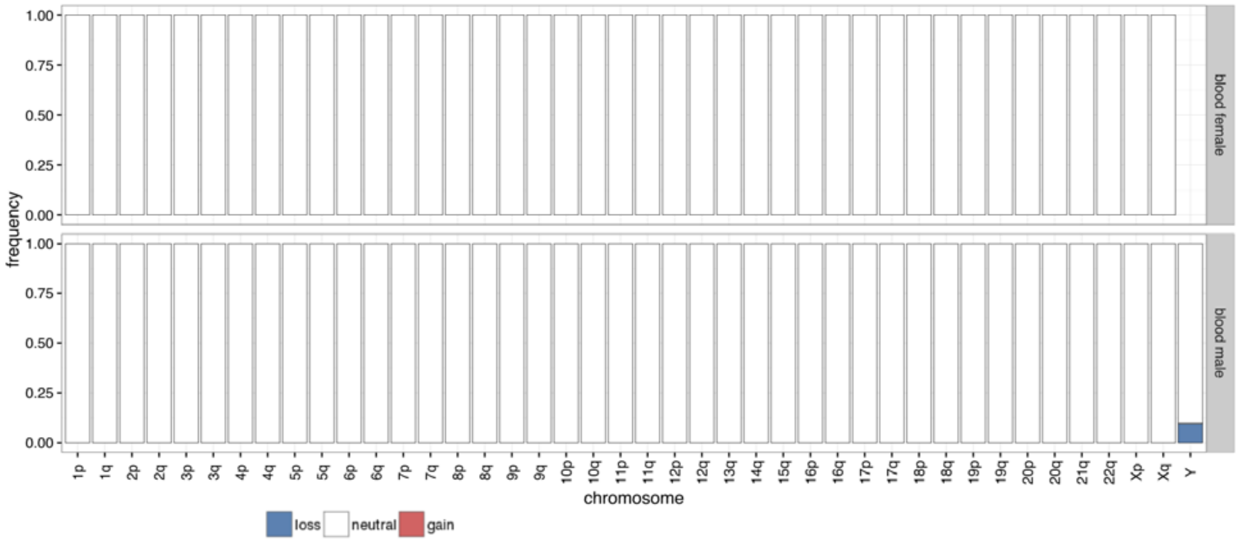

Supplementary Figure S2. Validation using PCR amplification. Status of chromosome Y in tumors and patient-matched normal samples is shown for individual male subjects of the validation set. The PCR amplification values are normalized and summarized by their median in each sample. Individuals affected by somatic LOY are shown in blue

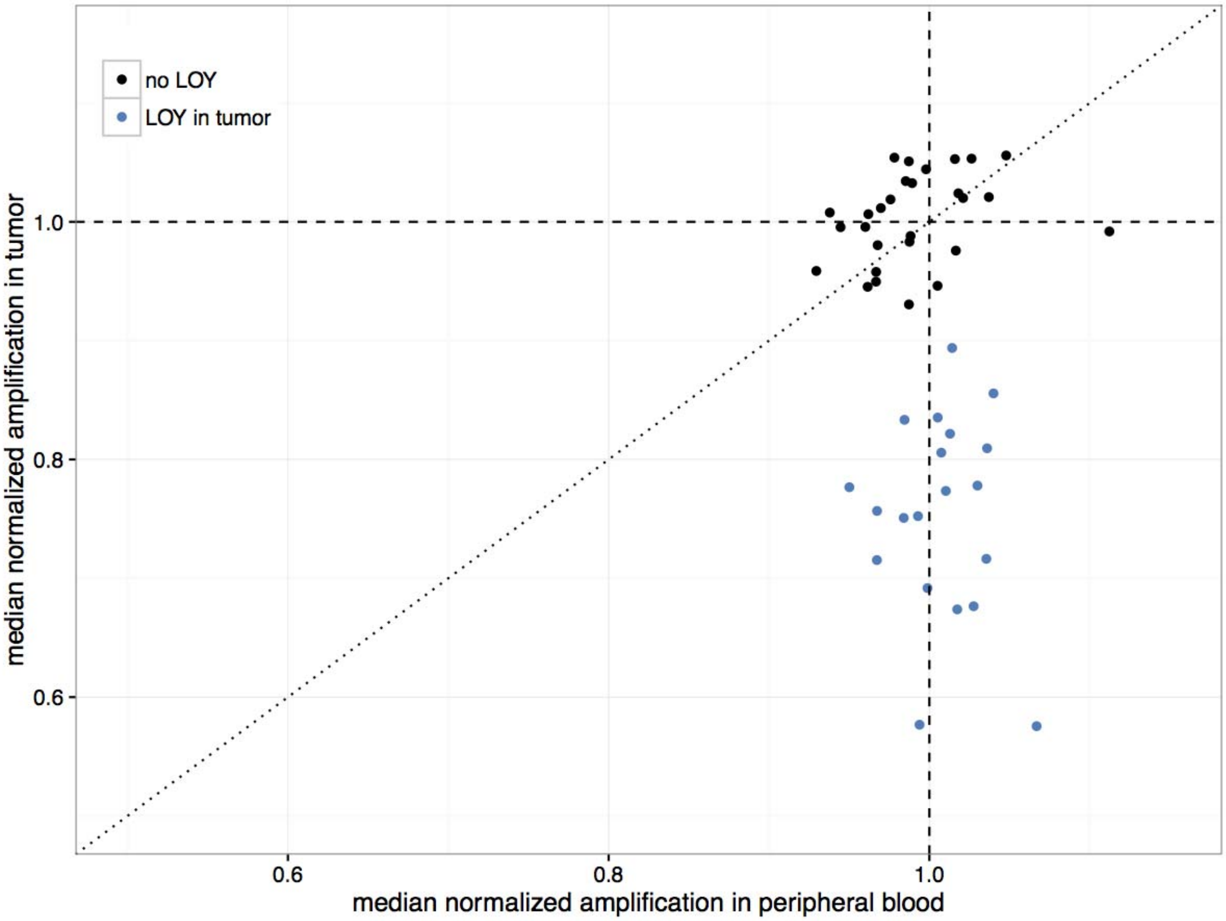

Supplementary Figure S3. Somatic LOY Y-linked genes down-regulation from array-based expression experiments. The proportion of cells with Y loss was estimated by the PCR amplification values.

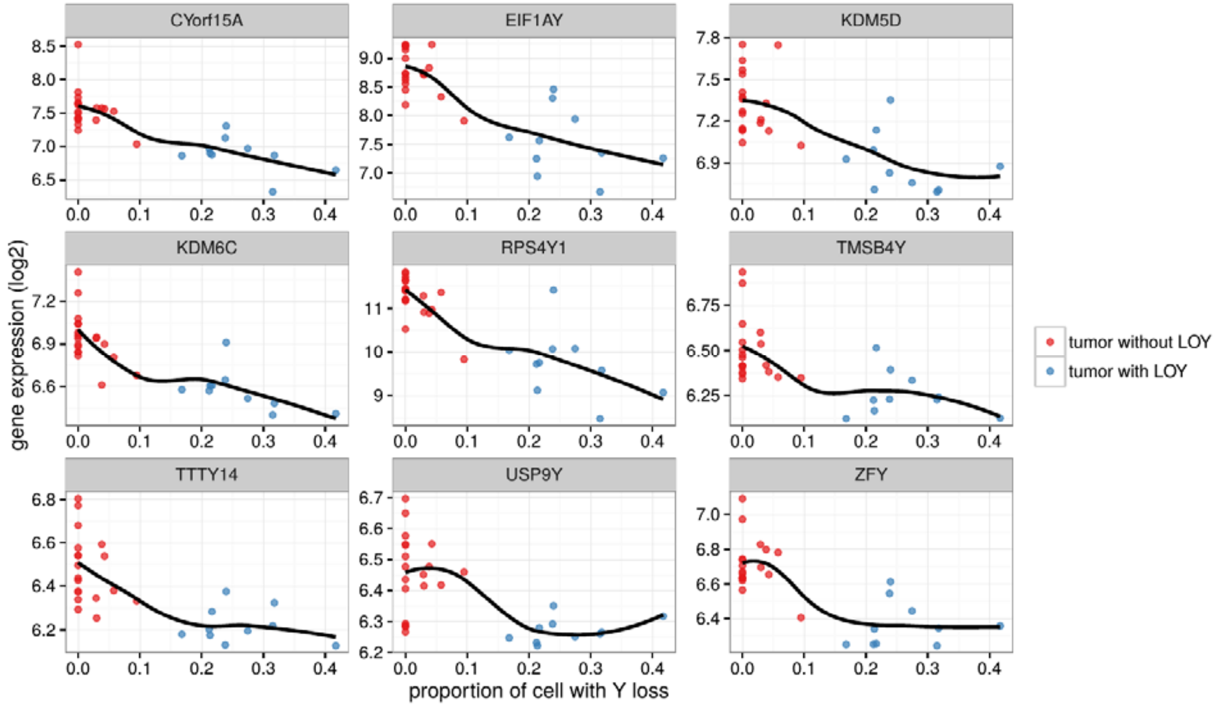

Supplementary Figure S4. Copy number aberrations of sex chromosomes and genomic status of X-linked epigenetic modifying genes in tumors of female and male patients. Nearly half of the female tumors harboring somatic LOX are also affected by mutations of *KDM5C*

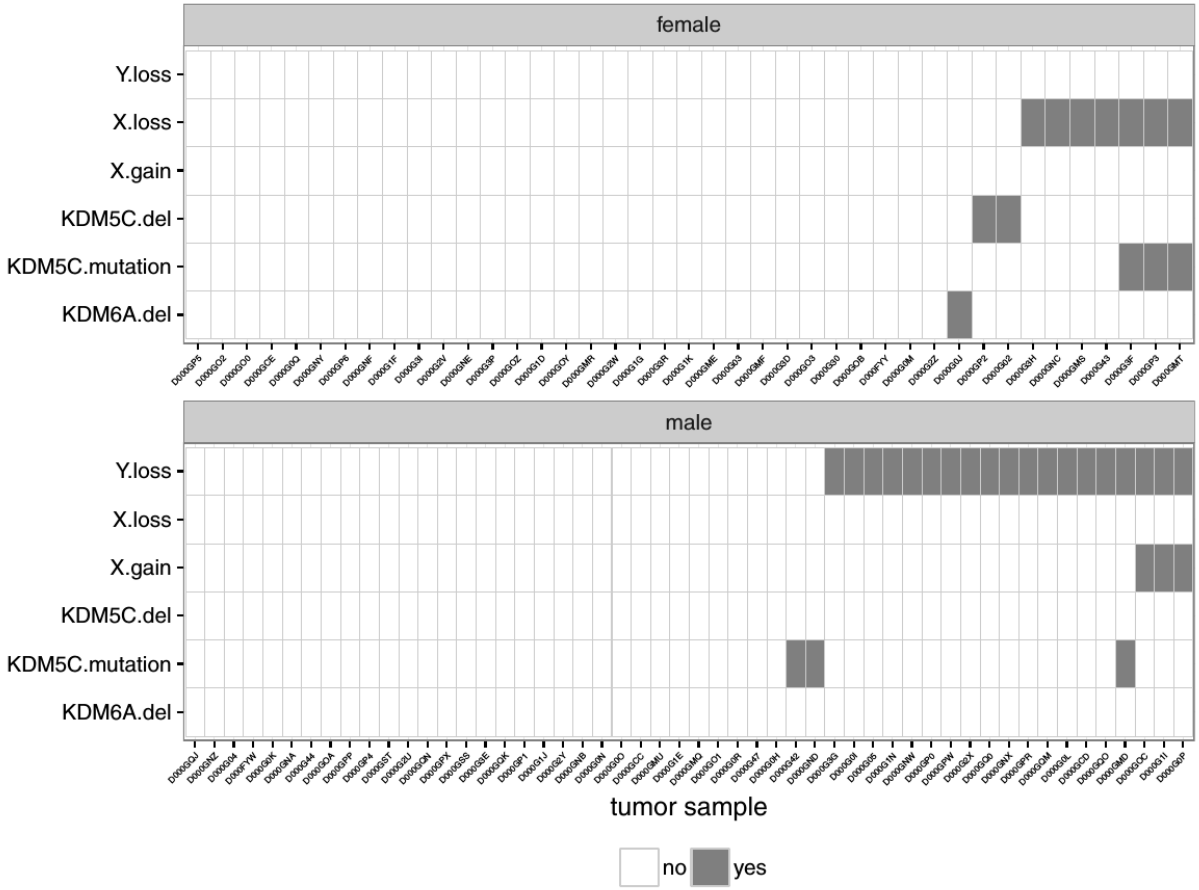

Supplementary Figure S5. Detecting loss of Y. The main Gaussian, fitted on the median normalized coverage, is used to detect significant loss/gain. Each male sample, blood and tumor, is colored according to its loss/gain status.

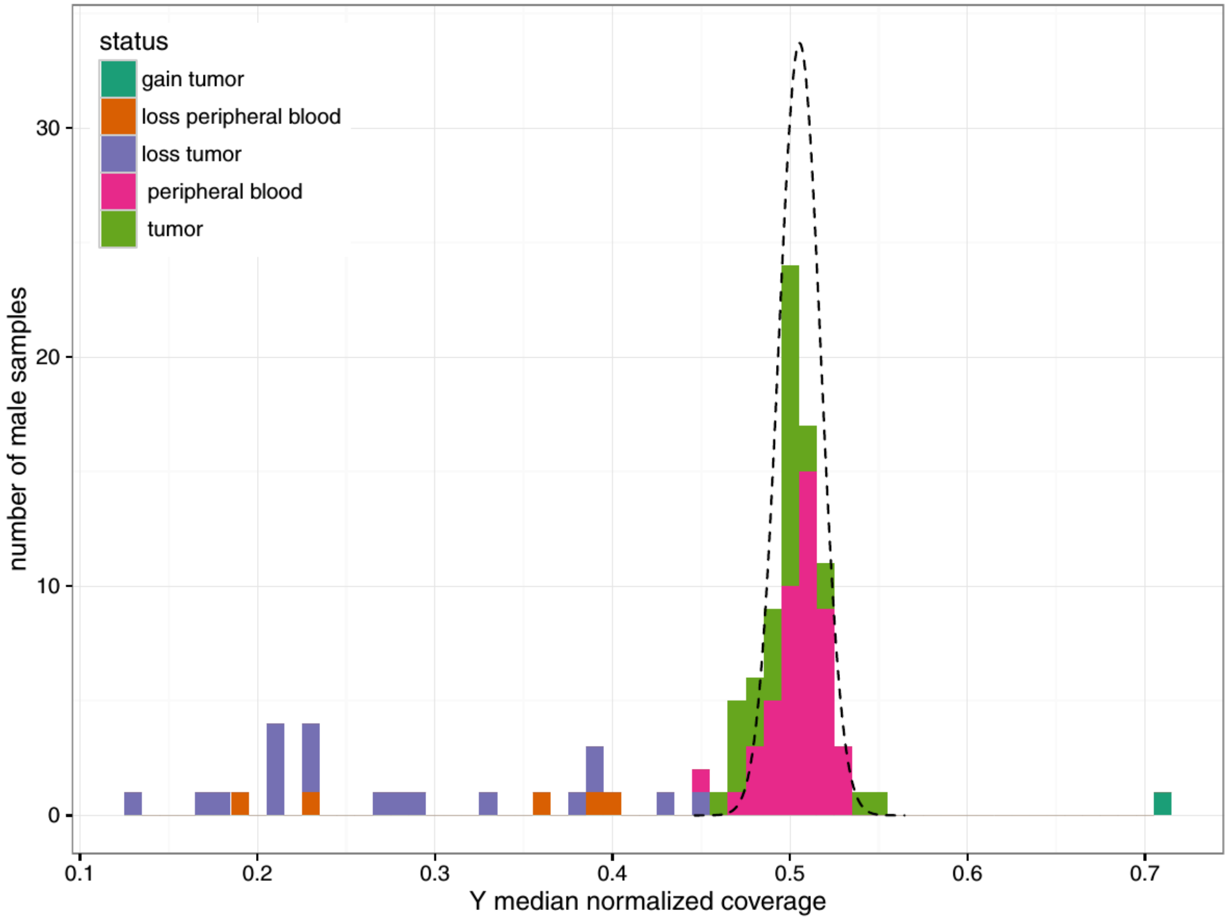

Supplement: Supplementary Information [file srep44876-s1.pdf]
